# Clinical Presentations and Predictors of In-Hospital Mortality in Illicit Drug Users in the New Psychoactive Substances (NPS) Endemic Era in Taiwan

**DOI:** 10.3390/toxics10070386

**Published:** 2022-07-12

**Authors:** Hsin-Tzu Yeh, Hsien-Yi Chen, Sung-Wei Liu, Te-I Weng, Cheng-Chung Fang, Jiun-Hao Yu, Yen-Chia Chen, Yu-Jang Su, Shi-Ying Gao, Chih-Chuan Lin

**Affiliations:** 1 Department of Emergency Medicine, Linkou Chang Gung Memorial Hospital, Taoyuan 333, Taiwan; yeh7504@gmail.com (H.-T.Y.); hshychen@gmail.com (H.-Y.C.); s78092359@cgmh.org.tw (S.-Y.G.); 2 College of Medicine, Chang Gung University, Taoyuan 333, Taiwan; 3 Department of Emergency, Hualien Tzu Chi Hospital, Buddhist Tzu Chi Medical Foundation, Tzu Chi University, Hualien 970, Taiwan; 101324115@gms.tcu.edu.tw; 4 Institute of Medical Sciences, Tzu Chi University, Hualien 970, Taiwan; 5 Department of Emergency Medicine, National Taiwan University Hospital and College of Medicine, National Taiwan University, Taipei 100, Taiwan; wengtei2@ntu.edu.tw (T.-I.W.); conrad@ntu.edu.tw (C.-C.F.); 6 Forensic and Clinical Toxicology Center, National Taiwan University Hospital and College of Medicine, National Taiwan University, Taipei 100, Taiwan; 7 Institute of Forensic Medicine, College of Medicine, National Taiwan University, Taipei 100, Taiwan; 8 Department of Emergency Medicine, China Medical University Hospital, Hsinchu 300, Taiwan; flykingyu@gmail.com; 9 Emergency Department, Taipei Veterans General Hospital, Taipei 11217, Taiwan; ycchen4@gmail.com; 10 Department of Emergency Medicine, School of Medicine, National Yang-Ming Chiao Tung University, Taipei 11217, Taiwan; 11 Department of Emergency Medicine, School of Medicine, National Defense Medical Center, Taipei 114, Taiwan; 12 Emergency Department, Mackay Memorial Hospital, Taipei 104, Taiwan; yjsu.md@msa.hinet.net; 13 Poison Center, Mackay Memorial Hospital, Taipei 104, Taiwan; 14 Department of Medicine, Mackay Medical College, New Taipei City 251, Taiwan; 15 MacKay Junior College of Medicine, Nursing, and Management, New Taipei City 251, Taiwan

**Keywords:** new psychoactive substance, mortality, prediction

## Abstract

Predictors of mortality in illicit drug users involving Novel Psychoactive Substances (NPS) and multiple substances have not been elucidated. We aimed to define predictors of mortality in the NPS endemic era’s illicit drug users to strengthen patient care in emergency treatment. This was a retrospective study. LC-MS/MS-confirmed positive illicit drug users who visited the emergency departments (ED) of six medical systems were enrolled. Demographic information, physical examinations, and laboratory data were abstracted for mortality analysis. There were 16 fatalities in 355 enrolled patients. The most frequently used illicit drugs were amphetamines, followed by opioids, cathinones, and ketamine. The most frequently detected cathinones among the 16 synthetic cathinones were eutylone, followed by mephedrone. The combined use of cathinones and ketamine was most commonly observed in our results. Univariate analysis revealed that the mortality patients were older, with deep coma, faster heart rate and respiratory rate, lower blood pressures and O_2_ room air saturation, more seizures, abnormal breath sounds, and had urine incontinence compared to the survivor patients. The mortality patients also had acute kidney injury, higher potassium, blood sugar, liver function test, and lactate level. The results of multiple logistic regression demonstrated that SBP < 90 mmHg, dyspnea, blood sugar > 140 mg/dl, and HCO_3_ < 20.6 mmHg were independent predictors of in-hospital mortality. Regardless of the pattern of the use of illicit drugs, the predictors allow for risk stratification and determining the optimal treatment.

## 1. Introduction

Over the past decade, there has been a worldwide increase in the opportunity to use and consume Novel Psychoactive Substances (NPS), which are synthetic alternatives to illicit drugs of abuse [1]. As defined by United Nations Office on Drugs and Crime (UNODC), NPS are substances not under international control but may pose a public health threat similar to substances under international control. NPS are easy to obtain, inexpensive, and not detected by standard toxicology screens [2]. The number of intoxicated people presenting with emergencies is increasing [3]. According to the 2019 World Drug Report, as of December 2020, UNODC identified a total of 1047 NPS [4]. In contrast to the effects of NPS at the population level, different NPS can be quite harmful at the individual level, with toxicology cases of single substances showing harmful effects, including death, because of their use [4].

There have been three major waves of drug epidemics in recorded history in Taiwan. Each wave involved different types of drugs tackled with different policies and measures. Since the early 2000s, club drugs, including methamphetamine, MDMA, ketamine, and flunitrazepam (also known as FM2 in Taiwan), have become popular in local rave parties and dance clubs. Some club drugs, such as ketamine, are now more familiar to the public as NPS [5]. Moreover, drug use is gradually shifting from traditional drugs to NPS in Taiwan. Ketamine remained the most popular NPS in Taiwan till 2016.

From 2017 onward, synthetic cathinones (such as mephedrone and MEAPP) have replaced ketamine as the most predominant NPS and deserve further attention in Taiwan for their abuse liability [6]. A recent clinical study reported six patients with “instant coffee sachets” which contained a mixture of illicit psychoactive substances, including amphetamines, ketamine, or cathinones [7]. The latest study in Taiwan associated with NPS was the “Taiwan Emergency Department Drug Abuse Surveillance (TEDAS)” project [8]. This observational study included collecting and analyzing urine samples and assessing the clinical presentation of patients from 79 emergency departments (EDs) across Taiwan. According to their result, the most frequently detected drug was methamphetamine/amphetamine, followed by synthetic cathinones, ketamine, and opioids. The main synthetic cathinones were mephedrone, ephylone, eutylone, and dibutylone. In addition to that, TEDAS also reported that nearly half of their enrolled patients used two or more two kinds of illicit drugs. The most common drug combination was cathinones plus ketamine and its analogs. In addition to that, TEDAS also reported that nearly half of their enrolled patients used two or more two kinds of illicit drugs. The most common drug combination was cathinones plus ketamine and its analogs.

Given the heterogeneous mix of components, abuse of NPS is expected to cause variable adverse health effects [9]. Synthetic stimulants may cause paranoia, hallucinations, and even seizure. Synthetic cannabinoids are associated with a wide range of side effects, including cardiovascular and respiratory complications, hemodynamic instability, renal injury, and cerebrovascular accidents. Synthetic hallucinogens could cause tachycardia, hypertension, hyperthermia, agitation, hallucinations, drowsiness, and even confusion. In Taiwan, according to the Institute of Forensic Medicine, Ministry of Justice, 250 cases of drug use-related death collected from 1361 medico-legal autopsy cases in 2018 shows that ketamine ranked fifth (*n* = 32, 12.8%) on the drug use-related death list. The other top four death-related drugs were: methamphetamine (*n* = 82, 32.8%), heroin (*n* = 65, 26.0%), flunitrazepam (FM2) (*n* = 35, 14.0%), and estazolam (*n* = 33, 13.2%) [6]. When facing the emergence of NPS and multiple substances use, we need to be familiar with clinical manifestations, management of mixed drug intoxications, prognosis, and clinical outcomes. Although a few reports have described the clinical course and outcomes following different kinds of NPS ingestion, predictors of serious complications and even mortality have not been elucidated in today’s complex situation [10,11,12]. This study aimed to define predictors of mortality in NPS endemic era’s illicit drug users to strengthen the accuracy and timeliness of the emergency treatment.

## 2. Materials and Methods

### 2.1. Study Design, Setting, and Selection of Participants

This was a retrospective study. From February 2019 to November 2019, liquid chromatography/mass spectrometer (LC-LM/MS) confirmed positive illicit drug users who visited the emergency departments (ED) of the following medical centers or hospitals were enrolled: Chang Gung Memorial Hospitals (including Linkou, Keelung and Kaohsiung CGMH), MacKay Memorial Hospital, National Taiwan University Hospital, Taipei Veterans General Hospital, Hualien Tzu Chi Hospital, and China Medical University. Enrolled patients were part of the recruit patients of the 2019 “Taiwan Emergency Department Drug Abuse Surveillance (TEDAS)” project. TEDAS project provided LC-LM/MS qualitative analysis of 110 kinds of illicit drugs (Supplementary Table S1), including non-NPS illicit drugs and NPS for the 79 EDs participating in the project across Taiwan. When ED patients were suspected of illicit drugs being used, urine samples were collected and sent to either of the two toxicological laboratories, the Forensic and clinical toxicology center of National Taiwan University and Taipei Veterans General Hospital Department of Clinical Toxicology and Occupational Medicine.

This study is reported according to the Strengthening the Reporting of Observational Studies in Epidemiology (STROBE) guidelines [13].

### 2.2. Measurements

We designed a standardized abstraction form to collect all variables from electronic medical records retrospectively. The following variables were collected: patient’s demographics including age, gender, and date of admission, triage vital signs, Glasgow Coma Scale (GCS), and positive physical examination of any neurological, psychiatric, cardiovascular, respiratory, or other symptoms upon ED admission, laboratory variables, and outcomes. Laboratory variables include white blood count (WBC), hemoglobin (Hb), platelet count, biochemical markers such as creatinine, sodium (Na), potassium (K), aspartate transaminase (AST), alanine aminotransferase (ALT), creatine-phospho-kinase (CPK) and venous/arterial blood gas were all recorded. Lab results of the urine drug test from TEDAS were abstracted. The primary outcome was in-hospital mortality.

### 2.3. Statistics

All statistical analysis was performed using SAS statistical software version 7.1 (SAS Institute, Cary, NC, USA). For univariate analysis between groups, continuous variables were expressed as the Mann-Whitney U test or Student *t*-test. The categorical variables were indicated as the Chi-Square test or Fisher’s exact test. The univariate and multivariate logistic regression analyses were applied to analyze predictors influencing mortality in NPS intoxication. A *p*-value less than 0.05 was considered statistically significant.

## 3. Results

### 3.1. Patient Characteristics

A total of 355 patients were included in this study, 233 (62.9%) of whom were male, and 122 (37.1%) were female. The most frequently used illicit drugs were amphetamines, followed by opioids, cathinones, and ketamine. The most frequently detected cathinones among the 16 synthetic cathinones were eutylone, followed by mephedrone. The combined use of cathinones and ketamine was most commonly observed in our results. Supplementary Tables S2 and S3 described the details of the used drugs individually.

Table 1 describes the clinical manifestations and laboratory findings of the main drug classes in our study. The cathinones users were younger than amphetamines, opioids, and ketamine users. The cathinones users also had higher body temperature than amphetamines, opioids, and ketamine users. Regarding patients’ conscious levels, opioids, cathinones, and ketamine users had more comatose (GCS ≤ 8) patients. Regarding symptoms and signs, cathinones users experienced more palpitation, hallucination, agitation, and delirium than the other two kinds of drug users. Not surprisingly, opioid users had the smallest pupils. Regarding laboratory findings, cathinones, amphetamines, and ketamine users had higher hematocrit than opioid users. The higher hematocrit may reflect these patients were hemoconcentration or dehydrated. The cathinones users also had higher creatinine levels. The opioid users were more respiratory acidic because of the respiratory depression effects of opioids.

There were 16 fatalities for a mortality rate of 4.5% (Table 2). The mean age of all patients was 39.32 ± 15.86. The mortality patients (49.94 ± 18.8 years old) were older than the survivors (38.81 ±15.6 years old, *p* = 0.01). Those who died also had higher heart rates (127.19 ± 28.33 vs. 104.61 ± 25.53, respectively, *p* = 0.00) and faster respiratory rates compared to the survivors (23.13 ± 5.75 vs. 19.65 ± 2.98, *p* = 0.01). Lower blood pressures were observed among the mortality patients when they admitted to EDs (systolic blood pressure 105.38 ± 28.91 mmHg vs. 127.01 ± 26.78 mmHg, *p* = 0.0; diastolic blood pressure 69.5 ± 30.32 mmHg vs. 78.78 ± 19.23 mmHg, *p* = 0.01; and mean arterial pressure 81.46 ± 29.04 mmHg vs. 94.86 ± 20.39 mmHg, *p* = 0.01). Also, we found those who died had lower O_2_ room air saturation than the survivors (89.17 ± 8.78% vs. 95.5 ± 6.23%, *p* = 0.0054). There were more patients with deep coma (GCS ≤ 8) in the mortality group (9/16 vs. 59/339, *p* = 0.0008).

Regarding the clinical presentations, the most common symptoms/sings was agitation (*n* = 92, 26%). As well, 38 (11%) patients had dyspnea, 26 (7.3%) patients had hallucination, and 25 (7%) patients had sweating on the arrival at ED (Table 3). The mortality patients had more dyspnea (*p* < 0.00), seizure/status epilepticus (*p* = 0.00), abnormal breath sound (*p* = 0.00) and urine incontinence (*p* = 0.03) than the survivors.

### 3.2. Laboratory Results

Univariate comparisons of laboratory data were presented in Table 4. When comparing to the survivors, the mortality patients had less platelet count (193.44 ± 128.2 vs. 273.98 ± 98.32 10^3^/µL, *p* = 0.01), higher creatinine (2.06 ± 1.19 vs. 1.24 ± 1.46 mg/dL, *p* < 0.00), higher potassium (4.94 ± 1.21 vs. 3.78 ± 0.59 mEq/L, *p* < 0.00), higher blood sugar (181.13 ± 154.6 vs. 126.54 ± 52.77 mg/dL, *p* = 0.02), higher liver function test (AST 214 (76-2388) vs. 33 (21–62), *p* < 0.00; ALT 47 (19-131) vs. 24 (14–39) U/L, *p* = 0.012; total bilirubin 1.79 (0.8–3.5) vs. 0.6 (0.4–1) mg/dL, *p* = 0.00) and higher lactate (3.44 (2.71–9.24) vs. 2.23 (1.39–3.65) mmol/L, *p* = 0.01). The mean of all patients of pH, FiO_2_, PCO_2_ and HCO_3_ were 7.36 ± 0.1, 45.08 ± 33.11%, 41.5 ± 11.87 mmHg and 22.69 ± 5.28 mmol/L respectively. FiO_2_ (*p* = 0.00), PCO_2_ (*p* = 0.01) and HCO_3_ (*p* < 0.00) had significantly difference between the survivors and those who died.

Thus, in summary, those who died were with deep coma, faster vital signs, more effort in respiration, more oxygen supply, more acidemia, acute kidney injury, hyperglycemia, and higher lactate level, indicating their more critical illness.

### 3.3. Multiple Logistic Regression

**Figure 1:**
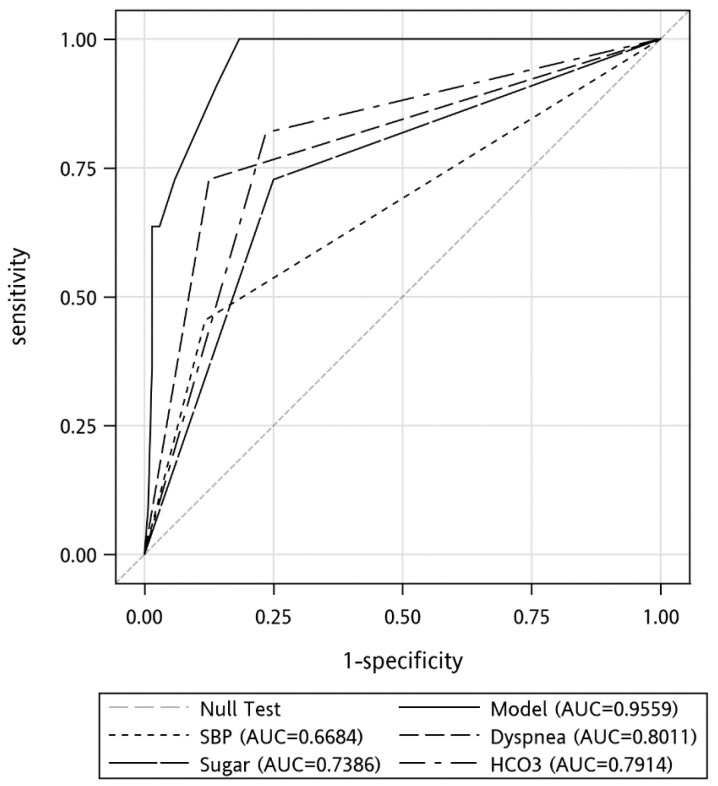
Receiver operating characteristic (ROC) curves for the prediction of the in-hospital mortality.

All the variables with a *p*-value < 0.1 were further analyzed through multiple logistic regression to identify independent predictors of in-hospital mortality. SBP < 90 mmHg (OR = 9.86), dyspnea (OR = 17.03), Sugar > 140 mg/dL (OR = 10.27) and HCO_3_ < 20.6 (OR = 13.35) mmHg was variable that remained in the model after logistic regression (Table 5). We compared the area under the receiver of operating characteristics (ROC) curve of these predictors (Figure 1). The areas under the curve of these predictors were: SBP < 90 mmHg, 0.67; dyspnea, 0.80; Sugar > 140, 0.74; HCO_3_ < 20.6, 0.79. The sensitivities, specificities, positive predictive value, negative predictive value, and accuracies of the above variables in predicting in-hospital mortality were 1, 0.82, 0.31, 1, and 0.83, respectively.

### 3.4. Drugs Used in the Mortality Patients

The drugs used in the mortality group were as below. The patients of single-use of drugs were morphine (*n* = 5), methamphetamine (*n* = 4), meta-chlorophenyl piperazine (*n* = 2), and 4-chloromethcathinone (*n =* 1). The patients of multiple use of drugs were methamphetamine + morphine+ codeine (*n* = 1), norketamine + morphine (*n* = 1) and N-ethyl pentylone + methamphetamine (*n* = 1). Four of sixteen were detected both with traditional illicit drugs and NPS.

## 4. Discussion

To the best of our knowledge, this study was the first cohort focused on clinical presentations and predictors of in-hospital mortality in illicit drug users in the NPS endemic era in Taiwan. Our results showed that the patients with higher heart rates, faster respiratory rates, lower systolic/diastolic/mean blood pressure, or GCS ≤ 8 were highly associated with in-hospital mortality. Lower platelet count, more severe renal injury with higher potassium level, higher blood sugar, more severe hepatic injury, and higher lactate acid level were also significantly related to in-hospital mortality. The independent predictors of in-hospital mortality were SBP < 90 mmHg, sugar > 140 mg/dL, dyspnea and HCO_3_ < 20.6 at the triage.

The overall mortality rate in our study was 4.5%. However, in French, 800 cases of NPS-related abuse or somatic complications were reported to the French Addict vigilance Network (DRAMES survey), including 71 fatal cases (9%) between 2009 and 2017 [3]. There were 3.2 NPS-related deaths per million inhabitants of countries reported in 2017 (range 0–19.8) and 4.9 per million inhabitants aged 15–64 (range 0–30.6) in five countries (Estonia, Finland, the UK, Sweden, and Turkey) [14]. Just as our study demonstrated, elderly-aged patients with substance abuse have higher mortality as described in the previous study [14]. Another retrospective study from the five Nordic countries: Denmark, Finland, Iceland, Norway, and Sweden, also showed many deaths numbers of addicts more than 45 years old [15].

Cardiovascular instability causing an increase in mortality in NPS intoxication patients was confirmed by our result of faster vital signs, but there have been little study into related topics. However, it is reasonable that patients with more severe cardiovascular effects such as hypotension or tachycardia would have a poorer prognosis, even death. Poor consciousness (GCS ≤ 8, 19% in this study) was also an important indicator of poor prognosis. These kinds of patients are prone to be intubated for airway protection. Sharon Essink et al. reported NPS intoxication symptoms in a prospective cohort that included patients with coma in seven cases (32%) and respiratory depression requiring mechanical ventilation in five cases (23%) [15]. Thus, when illicit drug users are in a deep coma, never hesitate to intubate the patients to have good supportive care.

The mortality patients had more severe metabolic acidosis. Metabolic acidosis indicated higher intoxication metabolites, direct cell injury, hypoxia, and possible increased lactate levels [16]. Clinically, acute metabolic acidosis decreases cardiac output, dilates arteries resulting in hypotension, alters oxygen delivery, decreases ATP production, causes a predisposition to arrhythmias, and impairment of the immune response [17]. The mortality patients also had significantly higher blood sugar levels. To our best knowledge, stress hyperglycemia describes a state of blood glucose deregulation during acute physiological stress, which is common in critically ill patients and appears to be a marker of disease severity [18,19]. It is an adaptive immune-neurohormonal response to physiological stress to increase metabolic substrates to struggling organs during a crisis [20]. In other words, if patients with NPS intoxication, higher hyperglycemia would be a poor sign for their prognosis due to more severe critical illness. Since blood glucose is easy and fast to obtain in the clinical setting, thus, blood glucose > 140 mg/dL may serve as an early warning sign in those severely ill illicit drug users. SBP < 90 mmHg indicated a poor prognosis, too. The most reliable explanation was that it caused shock status meaning poor perfusion and tissue hypoxia. Similarly, in another study, hypotension (mean arterial blood pressure of ≤ 59 mmHg) was identified as a predictor of mortality of elderly with acute poisoning in the ED [21]. Jayashree et al. reported hypotension at admission as the most significant predictor of death in children admitted to the ICU with acute intoxication [22].

In this study, we did not mainly focus on which type of NPS intoxication because many patients could present to the EDs with mixed or multiple drug intoxication. Instead, we attempted to identify patients with the highest risk of in-hospital mortality. Although the different characteristics of illicit drugs may vary in clinical presentations, it is quite impractical to treat patients until we confirm the culprit of illicit substances in the clinical setting. On the contrary, it is practical to know the red flag signs when patients are admitted to ED to treat them in priority and provide adequate, qualified, supportive care in advance and thus avoid poor outcomes. The four independent predictors may guide ED physicians to determine the initial severity of the patients with suspected illicit drug users and thus provide the intensive care treatment measurements.

In this study, we also described the main drug classes’ clinical manifestations, symptoms, signs, and laboratory findings. The cathinone users were younger, had higher body temperature, experienced more palpitation, hallucination, agitation and delirium, higher creatinine levels, and hemoconcentration/dehydration. Opioid users had the smallest pupils and were more respiratory acidic because of the respiratory depression effects of opioids. Different illicit drug classes had different clinical manifestations, as we demonstrated here. Therefore, their specific clinical manifestations provide their value in diagnosis. However, in clinical settings, nearly half of the illicit drug users used multiple substances and the typical clinical manifestations of each drug class may be less observable. Regrettably, we cannot determine each of their prognostic factors of mortality in this study because of the small patient numbers of each illicit drug classes we had.

Our study has some limitations. First, our study was retrospective. Therefore, the study design had inherent limitations such as recall bias. Although we made an effort to remain objective, this study was inevitably limited by missing data. Second, the concomitant illicit drug use pattern may differ across countries or regions; therefore, this study’s findings should be interpreted cautiously. However, the compensated cardiovascular functions, the stress blood sugar level, and metabolic acidosis with lacticaemia all point us to a severely ill patient.

## 5. Conclusions

Regardless of the pattern of the use of the illicit drugs, patients with deep coma, unstable vital signs, metabolic acidosis, and stress blood sugar levels were independent predictors in illicit drug users in the NPS endemic in Taiwan. The recognition of these associated factors allows for risk stratification and determining the optimal treatment.

## Figures and Tables

**Table 1:** The clinical manifestations of the main drug classes.

Variable	Drugs				*p*
	Amphetamines (*n* = 66)	Opioids (*n* = 61)	Cathinones (*n* = 36)	Ketamine (*n* = 27)	
Age	38.44 ± 11.07	48.43 ± 18.46	29.50 ± 12.70	40.33 ± 15.93	<0.01
Male, *n* (%)	51 (77.27)	42 (68.85)	23 (63.89)	23 (85.19)	0.19
Triage vital signs					
Body temperature (°C)	36.74 ± 0.91	36.73 ± 1.04	37.36 ± 1.44	36.73 ± 0.84	0.02
Heart rate (beats/min)	107.61 ± 25	100.03 ± 19.02	113.86 ± 30.25	103.70 ± 27.48	0.06
Respiratory rate (/min)	19.82 ± 2.68	19.87 ± 3.04	19.97 ± 4.38	19.27 ± 4.72	0.87
SBP (mmHg)	133.48 ± 27.84	123.05 ± 23.94	132.6 ± 24.74	131.38 ± 33.01	0.14
DBP (mmHg)	82.33 ± 19.92	78.31 ± 18.74	84.86 ± 20.92	76.85 ± 19.42	0.27
MAP (mmHg)	99.38 ± 21.41	93.22 ± 19.15	100.77 ± 20.82	95.03 ± 22.44	0.24
SpO_2_ (%)	94.65 ± 7.14	93.48 ± 6.84	95.78 ± 6.10	96.45 ± 4.38	0.32
GCS ≤ 8, *n* (%)	5 (7.58)	17 (27.87)	8 (22.22)	8 (29.63)	0.02
Symptoms/signs					
Palpitation	3 (4.55)	0 (0)	5 (13.89)	2 (7.41)	0.03
Chest pain	1 (1.52)	2 (3.28)	1 (2.78)	3 (11.11)	0.16
Dyspnea	5 (7.58)	10 (16.39)	6 (16.67)	2 (7.41)	0.31
Nausea/vomiting	1 (1.52)	6 (9.84)	5 (13.89)	1 (3.70)	0.07
Headache	1 (1.52)	3 (4.92)	3 (8.33)	0 (0)	0.22
Hallucination	9 (13.64)	1 (1.64)	5 (13.89)	1 (3.70)	0.04
Agitation/delirium	24 (36.36)	6 (9.84)	15 (41.67)	4 (14.81)	<0.01
Seizure/status epilepticus	4 (6.06)	4 (6.56)	4 (11.11)	2 (7.41)	0.81
Tremor	4 (6.06)	4 (6.56)	3 (8.33)	0 (0)	0.54
Pupil size	3 (3–3)	2.75 (2–3)	3 (2–3)	3 (2–3)	0.02
Abnormal breath sound (wheezing+ crackle)	3 (4.55)	9 (14.75)	3 (8.33)	1 (3.70)	0.16
Urine incontinence	2 (3.03)	6 (9.84)	0 (0)	0 (0)	0.05
Sweating	3 (4.55)	2 (3.28)	1 (2.78)	2 (7.41)	0.80
Myoclonus/rigidity	1 (1.52)	1 (1.64)	3 (8.33)	1 (3.70)	0.24
Laboratory findings					
WBC	10.07 ± 4.63	12.81 ± 7.3	12 ± 5.25	12.45 ± 11.38	0.15
Hct	41.59 ± 5.67	39.12 ± 6.08	42.32 ± 5.31±	40.97 ± 7.89	0.01
Platelet	260.53 ± 95.05	257.74 ± 112.54	322.5 ± 84.1	273.59 ± 125.07	0.01
Creatinine	1.12 ± 1.19	1.24 ± 1.1	1.56 ± 1.9	1.47 ± 1.46	0.03
Na (Sodium)	137.61 ± 5.9	137.06 ± 7.33	139.66 ± 5.06	136.64 ± 4.51	0.05
K (Potassium)	3.79 ± 0.75	3.92 ± 0.72	3.72 ± 0.68	3.95 ± 0.58	0.10
Cl (Chloride)	100.27 ± 7.06	103.67 ± 7.09	105.29 ± 6.45	102.36 ± 8.86	0.68
Sugar	129.98 ± 42.46	150.22 ± 101.44	128.69 ± 47.8	128.4 ± 42.71	0.72
CPK	257.5 (116.5–463.5)	182 (155–317)	282 (194–908)	472.5 (171–1819)	0.14
AST	62 (36–129)	37.5 (26.5–54.5)	19 (18–29)	30.5 (24–89)	0.01
ALT	30 (15–59)	27.5 (18–48)	17.5 (13–35)	26 (14–33)	0.29
Bilirubin total	0.57 (0.36–1.15)	0.58 (0.35–0.9)	0.69 (0.5–1.2)	0.51 (0.34–0.85)	0.66
Lactate	2.4 (1.37–6.01)	2.31 (1.65–3.2)	2.59 (1.63–6.98)	2.4 (1.28–5.14)	0.75
pH	7.37 ± 0.1	7.33 ± 0.11	7.4 ± 0.08	7.37 ± 0.09	0.04
PCO_2_	39.22 ± 10.07	47.2 ± 16.51	37.99 ± 10.88	37.97 ± 9.97	0.02
HCO_3_	22.37 ± 5.64	23.77 ± 5.88	22.63 ± 6.44	20.75 ± 5.2	0.21

**Table 2:** Characteristics of the illicit drug users on arrival at the ED of survivors and mortality patients.

Variable	Patients			*p*
	All (*n* = 355)	Survivor (*n* = 339)	Mortality (*n* = 16)	
Age	39.32 ± 15.7	38.81 ± 15.6	49.94 ± 18.8	0.01
Male, *n* (%)	233 (65.63)	223 (65.78)	10 (62.50)	0.10
Triage vital signs				
Body temperature (°C)	36.85 ± 1.1	36.82 ± 1.0	37.38 ± 2.0	0.59
Heart rate (beats/min)	105.63 ± 26.0	104.61 ± 25.5	127.19 ± 28.3	0.00
Respiratory rate (/min)	19.8 ±3.2	19.65 ± 3.0	23.13 ± 5.8	0.01
SBP (mmHg)	126.02 ± 27.2	127.01 ± 26.8	105.38 ± 28.9	0.01
DBP (mmHg)	78.36 ± 19.9	78.78 ± 19.2	69.5 ± 30.3	0.01
MAP (mmHg)	94.25 ± 21.0	94.86 ± 20.4	81.46 ± 29.0	0.01
SpO_2_ (%)	95.16 ± 6.6	95.5 ± 6.2	89.17 ± 8.8	0.01
GCS ≤ 8, *n* (%)	68 (19.15)	59 (17.40)	9 (56.25)	0.0008

Count data are expressed as numbers (percentage) and continuous values are expressed as mean ± SD.

**Table 3:** Clinical presentations of the illicit drug users on arrival at the ED of survivors and mortality patients.

Variable	Patients			*p*
	All (*n* = 355)	Survivor (*n* = 339)	Mortality (*n* = 16)	
Palpitation	19 (5.35)	18 (5.31)	1 (6.25)	0.59
Chest pain	15 (4.23)	14 (4.13)	1 (6.25)	0.51
Dyspnea	38 (10.70)	30 (8.85)	8 (50)	<0.00
Nausea/vomiting	22 (6.20)	22 (6.49)	0 (0)	1
Headache	13 (3.66)	13 (3.83)	0 (0)	1
Hallucination	26 (7.32)	25 (7.37)	1 (6.25)	0.71
Agitation/delirium	92 (25.92)	89 (26.25)	3 (18.75)	0.83
Seizure/status epilepticus	19 (5.35)	15 (4.42)	4 (25)	0.00
Tremor	18 (5.07)	16 (4.72)	2 (12.50)	0.19
Pupil size(left)	2.83 ± 1.1	2.79 ± 1.0	3.68 ± 2.2	0.31
Pupil size(right)	2.84 ± 1.1	2.79 ± 1.0	3.68 ± 2.2	0.32
Abnormal breath sound (wheezing + crackle)	23 (6.48)	18 (5.31)	5 (31.25)	0.00
Urine incontinence	16 (4.51)	13 (3.83)	3 (18.75)	0.03
Sweating	25 (7.04)	22 (6.49)	3 (18.75)	0.09
Myoclonus/rigidity	13 (3.66)	12 (3.54)	1 (6.25)	0.46

Count data are expressed as numbers (percentage) and continuous values are expressed as mean ± SD.

**Table 4:** Initial Laboratory data of the illicit drug users of survivors and mortality patients.

Variable	Patients			*p*
	All (*n* = 355)	Survivor (*n* = 339)	Mortality (*n* = 16)	
WBC (10^3^/uL)	11.77 ± 7.2	11.72 ± 7.1	12.65 ± 7.4	0.41
Hct (%)	40.58 ± 6.1	40.76 ± 6.0	36.72 ± 8.9	0.06
Platelet (10^3^/uL)	270.19 ± 101.1	273.98 ± 98.3	193.44 ± 128.2	0.01
Creatinine (mg/dL)	1.28 ± 1.5	1.24 ± 1.5	2.06 ± 1.2	<0.00
Na (Sodium, mEq/L)	137.9 ± 5.3	138.09 ± 5.0	133.94 ± 9.2	0.22
K (Potassium, mEq/L)	3.83 ± 0.7	3.78 ± 0.6	4.94 ± 1.2	<0.00
Cl (Chloride, mEq/L)	103.42 ± 5.7	103.51 ± 5.7	102.78 ± 6.0	0.97
Blood sugar (mg/dl)	129.42 ± 63.0	126.54 ± 52.8	181.13 ± 154.6	0.02
CPK (U/L)	223 (109–687)	223 (106–633)	241 (182–1336)	0.57
AST (U/L)	35.5 (22–84)	33 (21–62)	214 (76–2388)	<0.00
ALT (U/L)	24 (14–46)	24 (14–39)	47 (19–131)	0.01
Total bilirubin (mg/dl)	0.63 (0.4–1.1)	0.6 (0.4–1)	1.79 (0.8–3.5)	0.01
Lactate (mmol/L)	2.33 (1.45–3.68)	2.23 (1.39–3.65)	3.44 (2.71–9.24)	0.01
PH	7.36 ± 0.1	7.36 ± 0.1	7.35 ± 0.1	0.58
FiO_2_ (%)	45.08 ±33.1	41.98 ± 32.0	81.43 ± 24.1	0.00
PCO_2_ (mmHg)	41.5 ± 11.9	42 ± 11.9	33.67 ± 8.5	0.01
HCO_3_ (mmol/L)	22.69 ± 5.3	23.06 ± 5.1	16.87 ± 4.8	<0.00

Count data are expressed as numbers (percentage) and continuous values are expressed as mean ± SD or mean (Q1–Q3).

**Table 5:** Logistic model for predicting the probability of mortality of drug users.

Variable	β	Odds Ratio	95% Confidence Interval	*p*
Intercept	−1.8852			
SBP < 90	1.1441	9.86	(1.128, 86.129)	0.04
Dyspnea	1.4174	17.03	(2.947, 98.368)	<0.01
Sugar > 140	1.1644	10.27	(1.196, 88.117)	0.03
HCO_3_ < 20.6	1.2959	13.35	(1.774, 100.506)	0.01

## Data Availability

Not applicable.

## Supplementary Materials

The following supporting information can be downloaded at: https://www.mdpi.com/article/10.3390/toxics10070386/s1; Table S1: LC-MS/MS detection list of 110 drugs and metabolites. Table S2: Drugs use pattern. Table S3: The cathinones patients used.

## References

[1] Weinstein A.M., Rosca P., Fattore L., London E.D. (2017). Synthetic Cathinone and Cannabinoid Designer Drugs Pose a Major Risk for Public Health. Front. Psychiatry.

[2] Baumann M.H., Volkow N.D. (2016). Abuse of New Psychoactive Substances: Threats and Solutions. Neuropsychopharmacology.

[3] Batisse A., Eiden C., Peyriere H., Djezzar S. (2020). Use of new psychoactive substances to mimic prescription drugs: The trend in France. Neurotoxicology.

[4] United Nations Office on Drugs and Crime (UNODC) (2021). World Drug Report: Stimulants.

[5] Chang H.J. (1998). Health care systems in transition. II. Taiwan, Part II. The current status of HIV-AIDS in Taiwan. J. Public Health Med..

[6] Feng L.Y., Li J.H. (2020). New psychoactive substances in Taiwan: Challenges and strategies. Curr. Opin. Psychiatry.

[7] Chang H.-M., Tracy D.K., Huang M.-C., Pan C., Chen L.-Y. (2019). Psychiatric Profiles and Clinical Manifestations of Cathinone Users: Case Series of Analytically Confirmed Cathinone Use in Taiwan. Addict. Addict. Disord..

[8] Lin C.C., Weng T.I., Ng C.J., Shih C.P., Hsu J., Liao Y.C., Yang C.C., Fang C.C. (2022). Emergency department visits due to new psychoactive substances and other illicit drugs in Taiwan: Preliminary results of the Taiwan Emergency Department Drug Abuse Surveillance (TEDAS) project. Clin. Toxicol..

[9] Shafi A., Berry A.J., Sumnall H., Wood D.M., Tracy D.K. (2020). New psychoactive substances: A review and updates. Ther. Adv. Psychopharmacol..

[10] Flüchter P., Pajonk F.G. (2015). Treatment of intoxication with new psychoactive substances and methamphetamine. Med. Monatsschr. Pharm..

[11] Wilkins C. (2014). A critical first assessment of the new pre-market approval regime for new psychoactive substances (NPS) in New Zealand. Addiction.

[12] Mégarbane B., Oberlin M., Alvarez J.C., Balen F., Beaune S., Bédry R., Chauvin A., Claudet I., Danel V., Debaty G. (2020). Management of pharmaceutical and recreational drug poisoning. Ann. Intensive Care.

[13] von Elm E., Altman D.G., Egger M., Pocock S.J., Gøtzsche P.C., Vandenbroucke J.P. (2007). The Strengthening the Reporting of Observational Studies in Epidemiology (STROBE) statement: Guidelines for reporting observational studies. Bull. World Health Organ..

[14] López-Pelayo H., Vicente J., Gallegos A., McAuley A., Büyük Y., White M., Giraudon I. (2021). Mortality involving New Psychoactive Substances across Europe, 2016–2017. Emerg. Trends Drugs Addict. Health.

[15] Essink S., Nugteren-van Lonkhuyzen J.J., van Riel A., Dekker D., Hondebrink L. (2022). Significant toxicity following an increase in poisonings with designer benzodiazepines in the Netherlands between 2010 and 2020. Drug Alcohol Depend..

[16] Lee S.B., Kim D.H., Kim T., Lee S.H., Jeong J.H., Kim S.C., Park Y.J., Lim D., Kang C. (2019). Anion gap and base deficit are predictors of mortality in acute pesticide poisoning. Hum. Exp. Toxicol..

[17] Kraut J.A., Madias N.E. (2010). Metabolic acidosis: Pathophysiology, diagnosis and management. Nat. Rev. Nephrol..

[18] Marik P.E., Bellomo R. (2013). Stress hyperglycemia: An essential survival response!. Crit. Care Med..

[19] Scheen M., Giraud R., Bendjelid K. (2021). Stress hyperglycemia, cardiac glucotoxicity, and critically ill patient outcomes current clinical and pathophysiological evidence. Physiol. Rep..

[20] Mifsud S., Schembri E.L., Gruppetta M. (2018). Stress-induced hyperglycaemia. Br. J. Hosp. Med..

[21] Hu Y.H., Chou H.L., Lu W.H., Huang H.H., Yang C.C., Yen D.H., Kao W.F., Deng J.F., Huang C.I. (2010). Features and prognostic factors for elderly with acute poisoning in the emergency department. J. Chin. Med. Assoc..

[22] Jayashree M., Singhi S. (2011). Changing trends and predictors of outcome in patients with acute poisoning admitted to the intensive care. J. Trop. Pediatr..

