# Supplementary material for: Clinical Presentations and Predictors of In-Hospital Mortality in Illicit Drug Users in the New Psychoactive Substances (NPS) Endemic Era in Taiwan
Source: Toxics. 2022 Jul 12;10(7):386. doi: 10.3390/toxics10070386 (PMC9317329; doi:10.3390/toxics10070386)
Supplement: Supplementary file 1 [file toxics-10-00386-s001.zip › toxics-1791622-supplementary.pdf]

Table S1. LC-MS/MS detection list of 110 drugs and metabolites.

New psychoactive substances (NPS)			
**Drug types**	**Compounds**		
Synthetic Cannabinoids	JWH-018[1]<br>JWH-019[2]<br>JWH-022[3]<br>JWH-073[4]<br>JWH-081[5]<br>JWH-203[6]	JWH-250[7]<br>NM-2201[8]<br>UR-144[9]<br>XLR-11[10]<br>APINACA[11]<br>MDMB-CHMICA[12]	AB-FUBINACA[13]<br>AB-PINACA[14]<br>AMB-FUBINACA[15]<br>AB-CHMINACA[16]<br>5-Chloro-UR-144[17]
Phenethylamine	2C-C[18]<br>2C-I[19]<br>2C-E[20]<br>5-MAPB[21]<br>5-MAPDB[22]<br>5-APDB[23]<br>MDDMA[24]<br>MBDB[25]	6-methoxy methylone<br>N-Hydroxy-MDA[26]<br>25I-NBOMe[27]<br>25B-NBOMe[28]<br>4-Bromoamphetamine<br>4-Fluoroamphetamine<br>4-Chloroamphetamine	4-Fluoromethamphetamine<br>4-Chloromethamphetamine<br>4-Methoxymethamphetamine (MMA)<br>4-Methoxyethylamphetamine (PMEA)<br>N, N-Dimethylamphetamine
Synthetic cathinones	Ethylone<br>Butylone<br>Pentylone<br>Methylone<br>Mephedrone<br>Methedrone<br>Benzedrone<br>Ephylone<br>Methcathinone<br>Eutylone<br>D-Tertylone MDPV[29]<br>TH-PVP[30]<br>α-PPP[31]<br>α-PVP[32]<br>α-PHPP (PV8)[33]	α-PNP (PV10)[34]<br>3,4-MDPBP[35]<br>3,4-MDPHP[36]<br>4'-Methyl-α-PHP[37]<br>4-Chloro-α-PVP[38]<br>4-fluoro-α-PHP[39]<br>4-fluoro-α-PVP[40]<br>4-Methylpentedrone<br>Dimethylone (bk-MDDMA)<br>Dibutylone (bk-DMBDB)<br>4-Methylethcathinone (4-MEC)<br>4-Chloroethcathinone (4-CEC)	α-Ethylaminohexanophenone<br>3,4-Dimethylmethcathinone<br>N-Acetyl-3,4-MDMC[41]<br>4-Cl-N,N-DMC[42]<br>4-Chloromethcathinone (4-CMC)<br>4-Methyl-α-ethylamino-pentiophenone (4-MEAP)<br>4-Bromoethcathinone (4-BEC)<br>4-Bromomethcathinone (4-BMC)<br>4-Ethylmethylcathinone (4-EMC)<br>4-Fluoromethcathinone (4-FMC)
Phencyclidine-type substances	Ketamine<br>Norketamine	Methoxetamine<br>Deschloroketamine	4-MeO-PCP[43]<br>Deschloro-N-ethyl-ketamine
tryptamines	Bufotenin<br>5-MeO-DIPT[44]<br>5-MeO-MiPT[45]	5-MeO-DMT[46]<br>5-MeO-DALT[47]	α-Methyltryptamine (AMT)<br>N-Methyltryptamine (NMT)
Piperazines	BZP[48]<br>MBZP[49]<br>MT-45[50]	DBZP[51]<br>TFMPP[52]	p-Fluorophenylpiperazine<br>meta-Chlorophenylpiperazine
Other substances	Etizolam<br>Phenazepam<br>2-MAPB[53]	Methiopropamine<br>Diclazepam	2-Benzhydrylpyrrolidine (Desoxy-D2PM)
**Other illicit drugs**			
**Drug types**	**Compounds**		
Marijuana	11-Nor-9-carboxy-THC[54]		
Cocaine	Benzoylecgonine		
Opioids	Codeine	Morphine	6-Acetylmorphine
Amphetamine-type stimulants	Amphetamine<br>MDA[26]	Methamphetamine<br>MDMA[55]	

1. JWH-018: 1-pentyl-3-(1-naphthoyl)indole
2. JWH-019: (1-hexyl-1H-indol-3-yl)-1-naphthalenyl-methanone
3. JWH-022: 1-naphthalenyl[1-(4-penten-1-yl)-1H-indol-3-yl]-methanone
4. JWH-073: (1-butyl-1H-indol-3-yl)-1-naphthalenyl-methanone
5. JWH-081: (4-methoxy-1-naphthalenyl)(1-pentyl-1H-indol-3-yl)-methanone
6. JWH-203: 2-(2-chlorophenyl)-1-(1-pentyl-1H-indol-3-yl)-ethanone
7. JWH-250: 1-pentyl-3-(2-methoxyphenylacetyl) indole
8. NM-2201: naphthalen-1-yl 1-(5-fluoropentyl)-1H-indole-3-carboxylate
9. UR-144: (1-pentyl-1H-indol-3-yl)(2,2,3,3-tetramethylcyclopropyl)-methanone
10. XLR-11: (1-(5-fluoropentyl)-1H-indol-3-yl)(2,2,3,3-tetramethylcyclopropyl)methanone
11. APINACA: N-(1-Adamantyl)-1-pentyl-1H-indazole-3-carboxamide
12. MDMB-CHMICA: N-[[1-(cyclohexylmethyl)-1H-indol-3-yl]carbonyl]-3-methyl-L-valine, methyl ester
13. AB-FUBINACA: N-[(1S)-1-(aminocarbonyl)-2-methylpropyl]-1-[(4-fluorophenyl)methyl]-1H-indazole-3-carboxamide
14. AB-PINACA: (S)-N-(1-amino-3-methyl-1-oxobutan-2-yl)-1-pentyl-1H-indazole-3-carboxamide
15. AMB-FUBINACA: N-[[1-[(4-fluorophenyl)methyl]-1H-indazol-3-yl]carbonyl]-L-valine, methyl ester
16. AB-CHMINACA: N-[(1S)-1-(aminocarbonyl)-2-methylpropyl]-1-(cyclohexylmethyl)-1H-indazole-3-carboxamide
17. 5-Chloro-UR-144: (1-(5-chloropentyl)-1H-indol-3-yl)(2,2,3,3-tetramethylcyclopropyl)methanone
18. 2C-C: 2,5-dimethoxy-4-chlorophenethylamine
19. 2C-I: 2,5-dimethoxy-4-iodophenethylamine
20. 2C-E: 2,5-dimethoxy-4-ethylphenethylamine
21. 5-MAPB: 1-(benzofuran-5-yl)-N-methylpropan-2-amine
22. 5-MAPDB: 1-(2,3-dihydrobenzofuran-5-yl)-N-methylpropan-2-amine
23. 5-APDB: 2,3-dihydro-α-methyl-5-benzofuranethanamine
24. MDDMA: N,N,α-trimethyl-1,3-benzodioxole-5-ethanamine
25. MBDB: α-ethyl-N-methyl-1,3-benzodioxole-5-ethanamine
26. MDA: 3,4-methylenedioxyamphetamine
27. 25I-NBOMe: 2-(4-iodo-2,5-dimethoxyphenyl)-*N*-[(2-methoxyphenyl)methyl]ethanamine
28. 25B-NBOMe: 4-bromo-2,5-dimethoxy-N-[(2-methoxyphenyl)methyl]-benzeneethanamine
29. MDPV: methkylenedioxypyrovalerone
30. TH-PVP: 2-(pyrrolidin-1-yl)-1-(5,6,7,8-tetrahydronaphthalen-2-yl)pentan-1-one
31. α-PPP: α-pyrrolidinopropiophenone
32. α-PVP: α-pyrrolidinopentiophenone
33. α-PHPP (PV8): 1-phenyl-2-(1-pyrrolidinyl)-1-heptanone monohydrochloride
34. α-PNP(PV-10): 1-phenyl-2-(1-pyrrolidinyl)-1-nonanone
35. 3,4-MDPBP: 3',4'-methylenedioxy-α-pyrrolidinobutiophenone
36. 3,4-MDPHP: 3',4'-methylenedioxy-α-pyrrolidinohexiophenone
37. 4'-Methyl-α-PHP: 2-(pyrrolidin-1-yl)-1-(*p*-tolyl)hexan-1-one
38. 4-Chloro-α-PVP: 1-(4-chlorophenyl)-2-(1-pyrrolidinyl)-1-pentanone

39. 4-fluoro-PHP: 1-(4-fluorophenyl)-2-(pyrrolidin-1-yl)hexan-1-one
40. 4-fluoro-α-PVP: 1-(4-fluorophenyl)-2-(1-pyrrolidinyl)-1-pentanone
41. N-Acetyl-3,4-MDMC: N-acetyl-3,4-methylenedioxymethcathinone
42. 4-Cl-N,N-DMC: 4-chloro-N,N-dimethylcathinone
43. 4-MeO-PCP: 1-[1-(4-methoxyphenyl)cyclohexyl]-piperidine
44. 5-MeO-DIPT: 5-methoxy-N,N-diisopropyltryptamine
45. 5-MeO-MiPT: 5-methoxy-N-methyl-N-isopropyltryptamine
46. 5-MeO-DMT: 5-methoxy-N,N-dimethyltryptamine
47. 5-MeO-DALT: N,N-di allyl-5-methoxy tryptamine
48. BZP: 1-(phenylmethyl)-piperazine
49. MBZP: 1-methyl-4-(phenylmethyl)-piperazine
50. MT-45: 1-cyclohexyl-4-(1,2-diphenylethyl)-piperazine
51. DBZP: dibenzylpiperazine
52. TFMPP: trifluoromethylphenylpiperazine
53. 2-MAPB: N,α-dimethyl-2-benzofuranethanamine
54. THC: tetrahydrocannabinol
55. MDMA: 3,4-methylenedioxymethamphetamine

Table S2. The relationship between the drugs of self-report and the drugs detected by LC-MS/MS.

Drug of self-report	Drug detected by LC-MS/MS							
	Meth/amphetamine	Opioid	Cathinone	Ketamine	Marijuana	Cannabinoid	Tryptamine	Phenethylamine
Amphetamine (n=68)	56	5	18	14	1	1	1	3
Opioid (n=44)	21	34	3	5	0	1	0	0
Cathinone (n=35)	9	0	29	11	0	1	2	2
Ketamine (n=35)	21	2	17	20	0	0	0	1
Marijuana (n=6)	1	0	3	1	1	0	0	0
Unspecified illicit drug (n= 63)	32	19	26	20	0	0	0	3
